# Inference of Protein Complex Activities from Chemical-Genetic Profile and Its Applications: Predicting Drug-Target Pathways

**DOI:** 10.1371/journal.pcbi.1000162

**Published:** 2008-08-29

**Authors:** Sangjo Han, Dongsup Kim

**Affiliations:** 1 Department of Bio and Brain Engineering, KAIST, Daejeon, South Korea; 2 KAIST Institute for the Biocentury, KAIST, Daejeon, South Korea; King's College London, United Kingdom

## Abstract

The chemical-genetic profile can be defined as quantitative values of deletion strains' growth defects under exposure to chemicals. In yeast, the compendium of chemical-genetic profiles of genomewide deletion strains under many different chemicals has been used for identifying direct target proteins and a common mode-of-action of those chemicals. In the previous study, valuable biological information such as protein–protein and genetic interactions has not been fully utilized. In our study, we integrated this compendium and biological interactions into the comprehensive collection of ∼490 protein complexes of yeast for model-based prediction of a drug's target proteins and similar drugs. We assumed that those protein complexes (PCs) were functional units for yeast cell growth and regarded them as hidden factors and developed the PC-based Bayesian factor model that relates the chemical-genetic profile at the level of organism phenotypes to the hidden activities of PCs at the molecular level. The inferred PC activities provided the predictive power of a common mode-of-action of drugs as well as grouping of PCs with similar functions. In addition, our PC-based model allowed us to develop a new effective method to predict a drug's target pathway, by which we were able to highlight the target-protein, TOR1, of rapamycin. Our study is the first approach to model phenotypes of systematic deletion strains in terms of protein complexes. We believe that our PC-based approach can provide an appropriate framework for combining and modeling several types of chemical-genetic profiles including interspecies. Such efforts will contribute to predicting more precisely relevant pathways including target proteins that interact directly with bioactive compounds.

## Author Summary

Finding the specific targets of chemicals and deciphering how drugs work in our body is important for the effective development of new drugs. Growth profiles of yeast genomewide deletion strains under many different chemicals have been used for identifying target proteins and a common mode-of-action of drugs. In this study, we integrated those growth profiles with biological information such as protein–protein interactions and genetic interactions to develop a new method to infer the mode-of-action of drugs. We assume that the protein complexes (PCs) are functional units for cell growth regulation, analogous to the transcriptional factors (TFs) for gene regulation. We also assume that the relative cell growth of a specific deletion mutant in the presence of a specific drug is determined by the interactions between the PCs and the deleted gene of the mutant. We then developed a computational model with which we were able to infer the hidden activities of PCs on the cell growth and showed that yeast growth phenotypes could be effectively modeled by PCs in a biologically meaningful way by demonstrating that the inferred activities of PCs contributed to predicting groups of similar drugs as well as proteins and pathways targeted by drugs.

## Introduction

The collection of yeast deletion strains has enabled systematic genomewide functional analysis [1]. In addition, strain-specific molecular barcodes allow quantitative functional profiling of pooled deletion strains by using TAG oligonucleotide microarrays [1],[2]. One of several types of functional profiles, the chemical-genetic profile, expresses quantitative values of deletion strains' growth defects under a chemical. The compendium of chemical-genetic profiles of heterozygous and homozygous deletion strains under different chemicals has been successfully used for identifying direct target proteins of those chemicals [3],[4] as well as exploring their common mode-of-actions [5]. By integration of synthetic lethality profiles, the chemical-genetic profiles of homozygous deletion strains were also used to discover genes and pathways targeted by specific chemicals [6]. The chemical-genetic profiles in yeast are undoubtedly a useful resource to infer drug's action mechanism in human [7].

In the previous study, however, valuable biological information such as protein-protein and genetic interactions has not been integrated with chemical-genetic profiles in a single model for drug's target pathway prediction. Also, those profiles have not been yet modeled at the molecular level in terms of biological real entity such as protein complexes. As chemical-genetic profiles of many bioactive compounds are accumulating in many species including yeast, such real molecular entity-based modeling becomes essential to the molecular level understanding of phenotypes of the eukaryotic cell exposed to different chemicals. For example, that will allow us to infer drug's mode-of-action from chemical-genetic profiles more precisely than the previous model-free approach.

**Figure 1:**
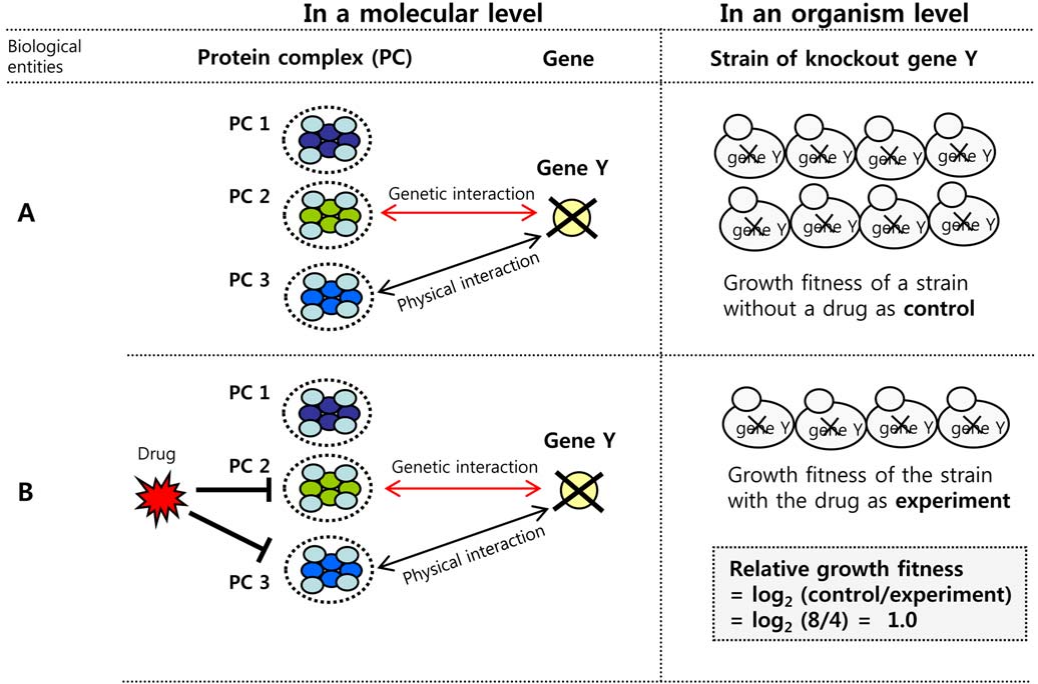
Model assumption on protein complexes as hidden factors underlying strain's fitness. Our model assumes that a protein complex (PC) is a functional unit to perform the biological processes in a cell, whose growth and survival is determined by the cooperative operation of a collection of PCs in a cell. (A) Suppose that a strain of Gene Y deletion has three PCs (PC 1, PC 2, and PC 3), and PC 2 and PC 3 are genetically and physically linked with Gene Y in a normal strain, respectively. (B) In general, genetic interactions have been involved in the genetic buffering in the redundant or parallel pathways, and the physical interactions tend to be involved in a sequential biological event through a serial pathway [49]. From this, the following scenario is plausible. When PC 2 and PC 3 are supposed to be the targets of a drug that blocks their functions, the inhibition of PC 2 by the drug will affect the growth of the strain of the gene Y deletion because some component of PC 2 cannot play a role of genetic buffer to some biological process involving Gene Y. In the extreme situation, the synthetic lethality or sickness will occur in the strain of gene Y deletion. In addition, the inhibition of PC 3 by the drug will also affect the growth of the strain because some component of PC 3 cannot interact with some component in the sequential biological process involving Gene Y anymore. Overall, the combined deleterious effects of the first neighbors PCs physically or genetically linked to gene Y will cause the unbalance of homeostasis of the strain. Consequently, the growth fitness of a strain treated with the drug (B) will be observed relatively lower than growth fitness of a strain treated with no drug (A).

In order to relate hidden biological activities of some real biological entities at the molecular level to the phenotypes of deletion strains at the organism level, we assume that the growth of a deletion strain is affected by hidden activities of protein complexes in a given condition, which leads to the observable population changes of the strain. This assumption comes from the molecular rationale of protein complexes for gene-to-phenotype relationships [8], by which similar sensitive phenotypes of deletion strains of genes comprising a protein complex were explained in various different conditions. It is plausible that protein complexes can be regarded as functional units for the phenotypes of a deletion strain. If we view the entire cell as a factory comprising various distinct machines efficiently connected for its productivity [9], genomewide protein complexes can be regarded as a collection of “cellular machines” connected with each other for optimal growth and survival of a cell (Figure 1).

**Figure 2:**
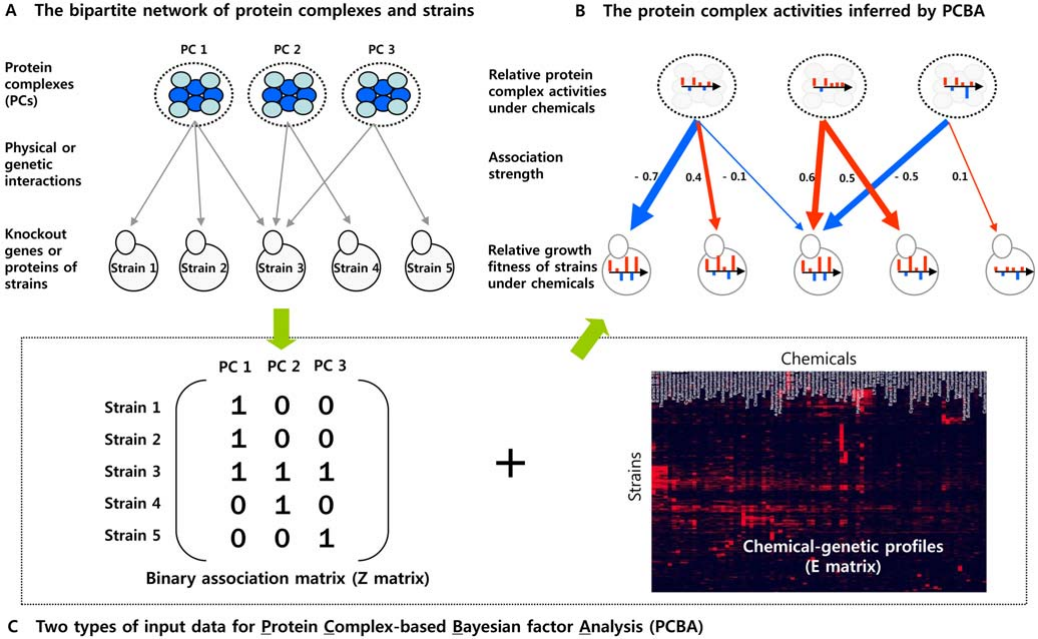
Procedures for inferring the hidden activities of a collection of protein complexes in a cell. (A) A bipartite network illustrating the first-order relationships between protein complexes (PCs) and strains they are associated with. The definitions of “protein complex”, “strains”, and “association” in the study are as follows: the first yeast comprehensive protein complexes reported by Gavin et al. [8] are used as a collection of “protein complexes” in a cell. The “strains” are defined as a collection of pooled deletion mutants released from Saccharomyces Genome Deletion Project [1]. The “association” is defined as the existence of physical or genetic interactions between at least one of components in PCs and knockout gene product of a strain. In bipartite network, we assume that the relative growth fitness of strains under different chemicals (called drugs or bioactive compounds in the text) is mainly caused by the deleterious associations of PCs and strains (Figure 1). (B) The bipartite PC-strain network reconstructed by applying PC-based Bayesian factor analysis (PCBA). The bar charts within dotted circles in the top of panel show the relative activities of PCs depending on chemicals inferred from our analysis. The bar charts within each strain in the bottom represent the relative growth fitness under different chemicals, which are used as observed data for our analysis. The thicknesses of arrows in the middle denote the association strength between PCs and strains inferred from our analysis. The colors of red and blue indicate “positive” and “negative” association, respectively. (C) It shows two types of input data for PCBA, one of which is a prior knowledge data of genetic and physical interactions in the left. It is represented in the form of matrix containing binary associations of each strain (row) to PCs (column) (called **Z** matrix in the text). If there was the association between the knockout gene of a strain *i* and at least one of components in a protein complex *j*, we set *z_ij_* = 1. Otherwise we set *z_ij_* = 0. The other is the chemical-genetic profiles representing relative growth fitness of pooled deletion strains under various chemicals. As the observed data for PCBA, it is shown in the right (called **E** matrix in the text).

Based on the assumption that each protein complex should play a proper role as well as communicate efficiently with each other for cell survival and adaptation in various treatments, we developed a Bayesian factor analysis model. Our model is similar to the network models developed in transcription regulation studies [10]–[13] (Figure 2). The basic idea of the model is that the observed growth fitness measurements of each strain are determined by combined effects of the activities of PCs in each cell in a given treatment. To implement this idea, the association relationship between deletion strains and PCs is necessary, which led us to construct the binary (0 or 1) association network between the knockout genes of strains and PCs based on their physical or genetic interactions (Figure 2C). Here, we assume that the relative growth fitness of strains by a chemical are mainly affected by deleterious interactions between the knockout-gene product of a strain and PCs which are physically or genetically linked (Figure 1).

By modeling chemical-genetic profiles in terms of protein complexes using physical and genetic interactions as a priori knowledge, we inferred hidden activities of a collection of PCs in each cell exposed to different chemicals. Based on those PC activities, bioactive compounds with similar mode-of-action were clustered together. It means that the binary association network in our model represents biologically meaningful relationship between knockout genes of strains and protein complexes as hidden factors. In addition, we showed that protein complexes with similar function were clustered together, which implies that the unknown functions of protein complexes can be predicted. Finally, we presented a new effective method to predict drug's target pathway using our PC-based model. For example, we were able to highlight target-protein, TOR1, of rapamycin as well as RUB1, UBA3, UBC12, and ULA1 related to protein neddylation as relevant biological pathway for cellular toxicity of camptothecin.

We believe that our protein complex-based approach can provide appropriate framework for combining and modeling several types of chemical-genetic profiles including interspecies. Such efforts will contribute to predict more precisely relevant pathway including target-proteins that interact directly with bioactive compounds.

## Results/Discussion

### Comparison of Complex-Based and Strain-Based Approaches in Terms of Prediction Common Mode-of-Actions of Drugs

**Figure 3:**
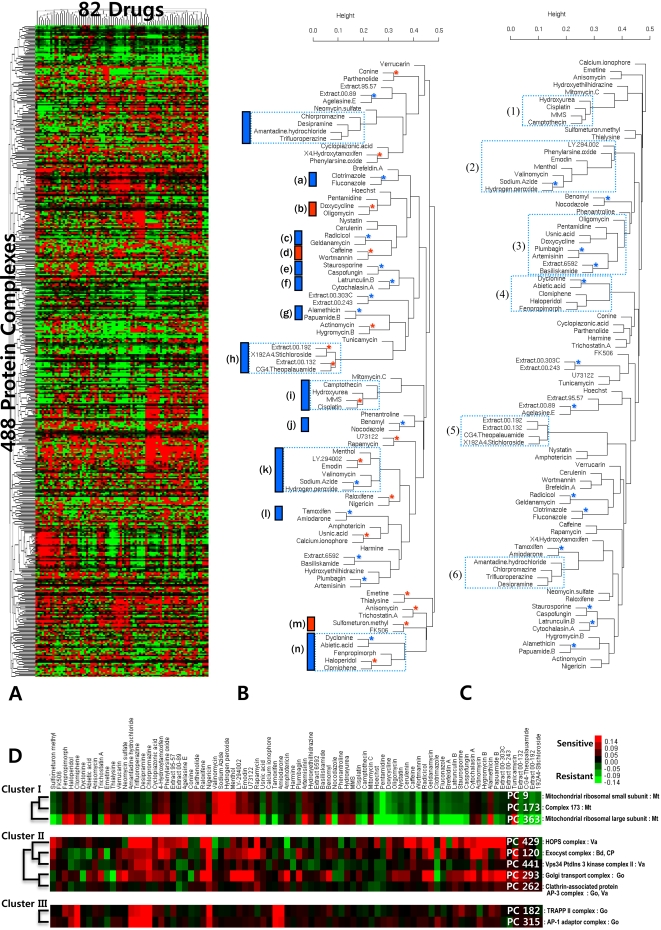
Clustering analysis of protein complex activities. (A) Bird eyes of two-dimensional hierarchical clustering analysis of protein complex activities (488 PCs by 82 drugs). (B) The PC-based hierarchical dendrogram of 82 drugs using relative activities of all of 488 protein complexes. The red star and red vertical bar indicates the group of drugs very closely clustered in the PC-based clustering but not in strain-based clustering using the same compendium [5]. The blue star and blue vertical bar represent the group of drugs very closely clustered in both clustering. The rectangular dashed line box shows the group of drugs clustered slightly differently in each clustering. The alphabets in parenthesis denote the groups of drugs with similar mode-of-action supported by literatures. (C) The strain-based hierarchical dendrogram of 82 drugs using the growth fitness of 3,418 strains with at least one measurement above Log_2_ ratio 0.5 as performed in the compendium paper [5]. (D) The examples of the clusters of PCs in similar biological functions. We selected three clusters which have at least two PCs with known function. The annotation in the right denotes “complex ID: complex name: complex localization”.

We applied our PC-based Bayesian factor model (see the details in Method Section) to the compendium of chemical-genetic profiles (∼4,800 haploid deletion strains, 82 chemicals) generated in the recent study [5]. This allowed us to infer the hidden activities of 488 PCs in 82 different chemicals (Figure 3A and Figure S3).

To compare our model-based approach with the previous strain-based approach for predicting common mode-of-action of drugs using the same compendium, we first performed hierarchical clustering of the inferred PC activities and strains fitness itself (Figure 3, see the details in Method section). For fair comparison, two different dendrograms of 82 drugs should be validated against the gold standard drug-drug associations in the cellular context of Yeast. However, such reliable data were not available enough to measure performance of two dendrograms quantitatively. Therefore, we marked the common clusters and different clusters on the two dendrograms (Figure 3B and 3C) and surveyed their literature evidences, which were categorized as follows:

#### Common Mode-of-Action of Drugs Supported by Both Complex-Based and Strain-Based Clustering

Many drugs were similarly closely clustered together in both dendrograms (see the blue star in Figure 3C). Among them, common mode-of-action of drugs in nine clusters, **(a)**, **(c)**, **(e)**, **(f)**, **(g)**, **(h)**, **(j)**, **(l)**, **and (n)**, have been already confirmed by the literature survey or experiments in the previous paper of releasing the compendium [5].

#### Common Mode-of-Action of Drugs Differently Supported by Complex-Based and Strain-Based Clustering

***Cluster (b).*** Doxycycline inhibits mitochondrial enzyme synthesis leading to a lack of oxidative ATP synthesis and so to proliferation inhibition in human leukemic cells [14], while oligomycin inhibits the oxidative ATP synthesis directly in *Saccharomyces cerevisiae* [15]. The complex-based cluster (b) reflects such physiological effects but strain-based cluster (3) does not.

***Cluster (d).*** Wortmannin and caffeine physiologically inhibit the signaling of phosphatidylinositol 3-kinase (PI3K)-related protein kinases, *TEL1* and possibly *MEC1* by regulating the level of inositol pyrophosphates, decreasing telomeric length and leading to cell death [16]. In aspect of regulating telomeric length, a recent paper suggests that rapamycin toxicity does not correlate with inositol pyrophosphate levels on cell death, and also targets of the rapamycin, *TOR1/2* may participate in the regulation by inositol pyrophosphates of vesicular endocytosis [16]. It is supported by *TOR2* localization to membranes of yeast vacuoles [17] and of *TOR1* to the plasma membrane of yeast [18] . The common molecular action of wortmannin and caffeine on cell death in *Saccharomyces cerevisiae* was represented in complex-based cluster (d), but not in the strain-based clustering.

***Cluster (h).*** The antifungal bioactive compound in extract 00-192, derived from a sea cucumber from the Commonwealth of Dominica, is identical to stichloroside as well as the antifungal compound in extract 00-132, derived from an Indonesian marine sponge, is identical to theopalaumide [5]. The stichloroside and theopalaumide do not share structural features. Nevertheless, the drug-resistant mutant study suggests that they share a common mode-of-action in yeast [5]. In aspect of functional classification of natural products, complex-based cluster (h) in our approach showed more sensitive result than strain-based cluster (5) by specifying compounds with antifungal activities in each crude extract.

***Cluster (i).*** A recent paper suggests that loss of vacuolar H+ translocating ATPase (V-ATPase) activity leads to abnormal intracellular acidification, which facilitates the DNA damage mediated by well known DNA-damaging agents, cisplatin, methyl methanesulfonate (MMS) and hydroxyurea (HU) [19]. The cisplatin is known for DNA inter-and intra-cross linking agent generating the platinum-DNA adducts, the most significant DNA lesions [20]. MMS is a monofunctional DNA alkylating agent leading to a lethal lesion primarily by methylating DNA on N^3^-deoxyadenine [21]. While MMS and cisplatin themselves are potent damaging agents, HU and camptothecin are known for ribonuclease reductase inhibitor giving rise to stalled replication forks that are sensed by the cell as abnormal DNA structures [22], and for a specific inhibitor of type I DNA topoisomerase trapping the covalent complex formed between catalytically active enzyme and DNA in *Saccharomyces cerevisiae* [23] as well as inducing DNA breakage at replication forks [24], respectively. Both of complex-based clusters (i) and strain-based ones (1) showed such similar mode-of-action of the group of damaging drugs. However, complex-based cluster (i) seemed to be more reasonable than strain-based cluster (1).

***Cluster (k).*** LY294002 is a cell-permeable compound that acts as a potent and selective inhibitor of phosphatidylinositol 3-kinase (PI3K) [25], which also serves as the molecular target for emodin to suppress tumor cell migration [26]. Whereas strain-based cluster (2) did not group two drugs into the nearest neighbors, complex-based cluster (k) showed the improved clustering result.

***Cluster (m).*** Sulfometuron methyl, a sulfonylurea herbicide, blocks growth of bacteria, yeast, and higher plants by inhibition of acetolactate synthase, the first common enzyme in the biosynthesis of branched-chain amino acids, leucin, isoleucin and valine [27]. The immunosuppressant FK506 inhibits amino acid import by targeting the yeast amino acid permease family, *TAT1* and *TAT2* in the posttranscriptional level [28]. In particular, a role of *TAT1* in branched-chain amino acid uptake was reported [29],[30]. Taken together, the treatments of sulfometuron methyl and FK506 lead to the depletion of branched amino acid through blocking its biosynthesis and uptake in *Saccharomyces cerevisiae*, respectively. Our complex-based cluster (m) reflected such same physiological effect, but the strain-based clustering did not.

We found that the complex-based clusters showed more physiologically meaningful grouping of drugs compared with strain-based clusters in yeast. For example, sulfometuron methyl and FK506 are very different chemicals in their structures, by both of which the same physiological effect of branched amino acid depletion occurs in yeast. What enables PC-based clustering to group together drugs with similar cellular defect in yeast better than strain-based clusters? Possible reasons are as follows:

First, 52% of 1114 essential genes currently released in Saccharomyces Genome Deletion Project [1] are included in 488 protein complexes, while strain-based clustering cannot inherently use haploid strains of knock-out essential genes (Figure S1B). Furthermore, a protein complex is composed of 5.5 essential and 7.8 non-essential genes on average. In other words, on average 41% of genes involved in a protein complex are essential for cell survival (Figure S1A), which implies that protein complexes as hidden factors may reflect relevant features for cell survival.

Second, valuable biological information on physical and genetic interactions are used to associate protein complexes with haploid deletion strains in PC-based modeling. This integration of prior knowledge allowed us to reduce the large dimension of chemical-genetic profiles (4111 by 82) into relatively small dimension of chemical-PC profiles (488 by 82) in a biologically meaningful way. That process makes chemical-PC profiles represent relevant features of chemical-genetic profiles at the minimum information loss. Furthermore, noise reduction by reweighting of irrelevant information is achieved by the dimension reduction in our model. It is indirectly supported that PC-based clustering is more robust than strain-based clustering. For example, removal of 27% of 488 protein complexes with low values did not significantly influence the clustering result made by all of 488 protein complexes, whereas removal of 7% of 4111 strains with low values made different clustering result compared with all of 4111 strains (Figure S2).

Taken together, we claim that the binary associations of 488 PC and 4111 strains (binary association matrix or **Z** matrix in Figure 2) in our model reflect biologically meaningful relationships between the strains as the observed data and the protein complexes as the hidden factors. We believe that the physical and genetic interactions as a priori for constructing the association matrix of PCs and strains are appropriate in our model, and furthermore their associations only between the knock-out gene products of strains and their first-neighboring protein complexes are sufficient for the dimension reduction in a biologically meaningful way.

### Grouping of the Protein Complexes with Similar Cellular Process

In strain-based clustering of the compendium [5], deletion mutant strains with similar chemical sensitivities are clustered together as well, grouping functionally related genes. Similarly, the protein complexes with a similar function were also clustered together (Figure 3D). For examples, we selected three clusters with at least two protein complexes known for their functions. Their functional annotations of Gene Ontology were summarized in Table S1. Here, we describe more literature evidences for those clusters.

1. Two subunits of mitochondrial ribosome (PC 9 and PC 363) supports that the protein complexes of “cluster I” are obviously related to the protein synthesis within mitochondrion.
2. The “cluster II” represented the groups of protein complexes involved in ER-to-Golgi and sequentially Golgi-to-vacuole or Golgi-to-endosome vesicle transports. Especially, the Sec34/35 Golgi Transport Complex (PC 293) in “cluster II” is known for tethering factors that attach the vesicle to the destination organelle prior to fusion to retrograde vesicular trafficking within the Golgi apparatus [31]. Whyte et al. shows that such protein complex is related to the Exocyst complex (PC 120), known as hetero-oligomers tethering factor at the plasma membrane, by sequence homology, and also suggested that they might perform analogous roles in different membrane traffic steps [32].
3. The TRAPP II complex (PC 182) in “cluster III”, one of two forms in TRAPP complex, functions as the tethering factor at the Golgi. Whereas the other, TRAPP I complex regulates YPT1 related to the entry of cargo vesicles into the Golgi apparatus, TRAPP II complex (PC 182) regulates YPT31 and YPT32 controlling exit of the cargo from the trans-Golgi network, residing in trans-Golgi [33]. Similarly, AP-1 adaptor complex (PC 315), along with clathrin-coat protein, drives transport vesicles formation at the trans-Golgi network, whereas AP-2 adaptor complexes are principally targeted to the plasma membrane [34].

As shown in the above examples, the hierarchically clustered groups of protein complexes revealed insights into similar biological processes. In other words, unknown functions of protein complexes could be inferred based on those groups closely clustered. Nonetheless, it was preliminary investigation using simple hierarchical clustering method to highlight the biological essential features of protein complex activities. If chemical-PC profiles are applied to other clustering methods such as biclustering or network reconstruction methods, such results could be different in some cases as well as give more essential features on behaviors of protein complexes perturbed by drugs.

### Prediction of Relevant Biological Pathways Targeted by a Drug

**Figure 4:**
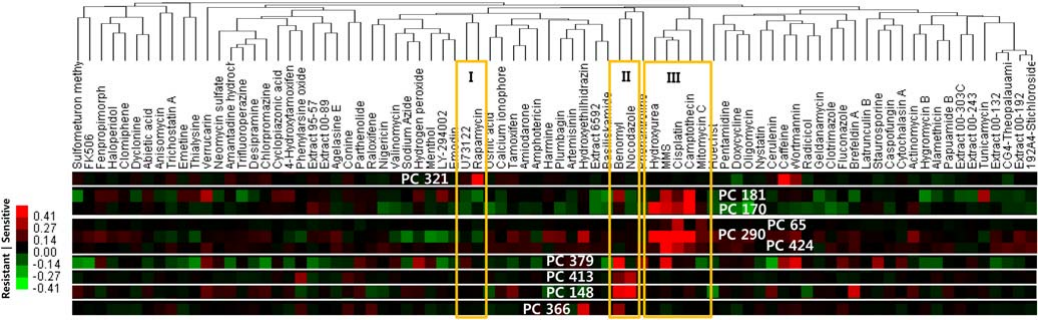
Significantly sensitive protein complexes to drugs known for their target pathway. The significance score of the effect of a compound on a protein complex was estimated using error function (see Method for details). When a significance score of a compound on a complex was less than a given threshold, and also that complex has relatively positive value of an activity, the complex was defined as the “sensitive complex” to that compound. For rapamycin in cluster I, there is only one sensitive complex, PC 321. There are four sensitive complexes, PC 379, PC 413, PC 148, and PC 366 to compounds in cluster II which are microtubule-poisons. Those DNA-damaging agents of cluster III have more than five sensitive complexes, PC 181, PC 170, PC 65, PC 290, and PC 424. The types of biological associations between each sensitive complex and their sensitive strains are available, and also GO analysis results of the set of those genes of such sensitive strains at http://pombe.kaist.ac.kr/CMA/ModeOfAction.pl.

**Figure 5:**
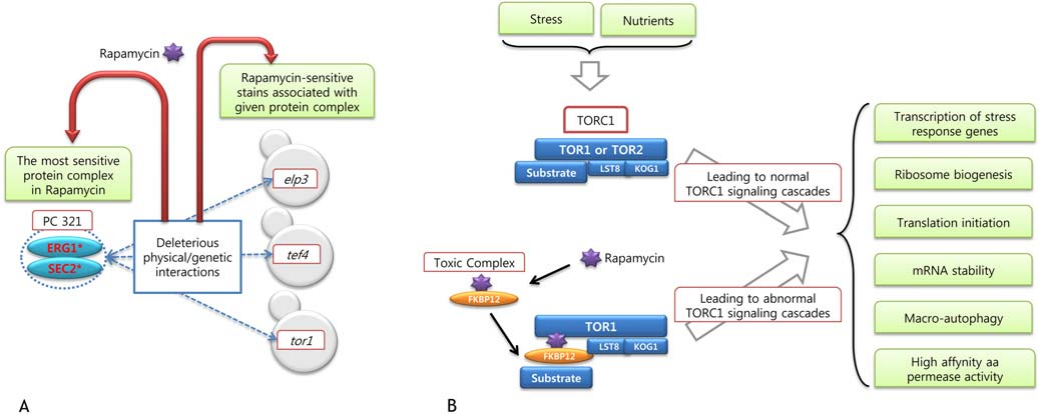
Target pathway of rapamycin. (A) The most sensitive protein complex to rapamycin, PC 321, is composed of ERG1 and SEC2, both of which are essential genes. Any of components in such complex may be physically or genetically associated with ELP3, TEF4, and TOR1 among gene products deleted in all the sensitive strains to rapamycin. According to model assumption, it can be interpreted as follows: Strains of complex-associated gene deletions have deleterious biological interactions with that complex. It may lead to decrease in the growth fitness of those strains in rapamycin. (B) Target of Rapamycin (TOR) pathway primarily regulated by Target of Rapamycin Complex 1(TORC1) with/without rapamycin. When the rapamycin is treated in a yeast cell, it binds FKBP12, forming toxic complex, which inhibits specifically TOR1, an essential component of TORC1. It gives rise to abnormal TORC1 signaling cascades related to broad biological functions, transcription, translation, mRNA stability and permeability.

**Figure 6:**
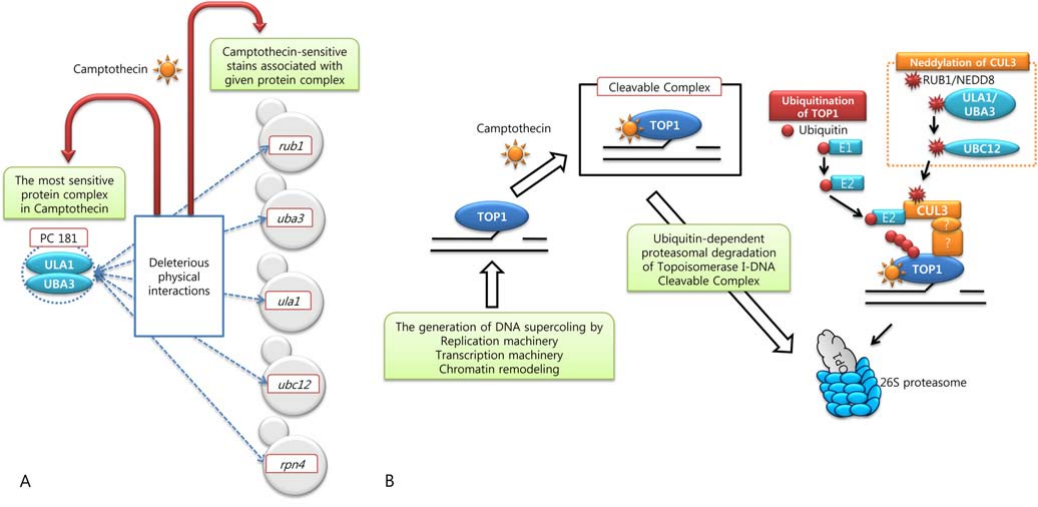
Target pathway of camptothecin. (A) The most sensitive protein complex to camptothecin, PC 181, is composed of ULA1 and UBA3, both of which are non-essential genes. Any of components in such complex may be physically associated with RUB1, UBA3, ULA1, UBC12, and RPN4 among gene products deleted in all the sensitive strains to camptothecin. (B) Our proposed model of neddylation-enhanced and ubiquitin-dependent proteasomal degradation of Topoisomerase I-DNA complex stabilized by camptothecin in yeast (details in the text). In this model, we suggest that RUB1-attachment of CUL3 may enhance the degradation of TOP1-cleavable complexes and, therefore, the blocking of RUB1-conjugation pathway contributes to significant increase of the level of camptothecin toxicity in cell growth as shown in PC 181-associated strains.

To find a drug-target pathway, we first selected significantly sensitive protein complexes to each drug (Figure 4), and then examined the biological processes of deletion genes of strains that were associated with such sensitive protein complexes in our model (see the details in the method; Table S2). This investigation of PC-constrained strains allowed us to highlight more relevant drug-target pathways than that of PC-free strains. In particular, it is clear in cases of camptothecin and rapamycin whose target-pathways are well known (Figures 5 and 6).

#### Rapamycin Targeting TOR1

Rapamycin and its analogues have a unique mode-of-action as follows: Rapamycin first binds to the 12-kDa immunophiln FK506-binding protein (FKBP12) and this complex inhibits the target of rapamycin (TOR) phosphoinositide-kinase (PIK)-related serine/threonine kinase conserved in all eukaryotes which is rapamycin target as well as subunit of TORC1 complex [35],[36] (Figure 5B). That complex is known for controlling growth in response to nutrients or stress by regulating translation, transcription, mRNA stability, ribosome biogenesis, nutrient transport and autophagy [36],[37]. Based on physical and genetic interactions with the most sensitive protein complex to rapamycin, PC 321, our PC-based approach suggests that three knock-out genes of elp3, tef4 and tor1 strains among all of 236 strains sensitive to rapamycin play important roles in rapamycin-target pathway (Figure 5A). These genes had no enriched Gene Ontology (GO) terms but one of them is rapamycin target-protein itself. It indicates that PC-constrained strains narrows down lots of rapamycin-sensitive genes involved in various biological pathways to a handful of genes that are important in the relevant pathway perturbed by rapamycin (Table S2).

#### Camptothecin Targeting Topoisomerase I

Camptothecin is a natural product of which Topoisomerase I (TOP I) is the only cellular target. TOP I is essential in higher eukaryotes, as they are required to relax DNA supercoiling generated by transcription, replication and chromatin remodeling. Despite their frequency throughout the genome, their cleavage intermediate steps (referred to as the “cleavable complexes”) are normally so transient that they are not detectable, but it is these complexes that are specifically and reversibly trapped by camptothecin [38]. In mammalian cancer cell, the degradation of such TOP I cleavable complex is known for being performed by the ubiquitin-proteasome system involving Cul3-based E3 ligase complex [39].

Based on physical interactions with the most sensitive protein complex to camptothecin, PC 181, our complex-constrained strains suggests that five knock-out genes of *rub1*, *uba3*, *ubc12*, *ula1*, and *rpn4* strains among all of 303 strains sensitive to camptothecin play important roles in camptothecin-target pathway (Figure 6A). Their enriched GO terms are described in Table S2. In particular, four of five genes selected are involved in protein neddylation (GO:0045116). In *S. cerevisiae*, RUB1 (called NEDD8 in human) among them is a ubiquitin-like post-translational modifier that is covalently linked to cullin (Cul)-family proteins in a manner analogous to ubiquitination [40]. In in vitro mammalian cell lines [41] and in vivo *S. pombe* [42], Rub1/NEDD8 attachments to cullin family members has been correlated with increased interaction of an S-ester-linked E2 with the E3 ligase, resulting in an activation of ubiquitination activity. In *S. cerevisiae*, conjugation of RUB1 is not essential for normal cell growth, but occurs selectively in a small set of substrates, CDC53, RTT101, and CUL3 [39]. Furthermore, it is suggested that such modification of cullin family by RUB1 conjugation might be functionally affiliated to the ubiquitin-proteasome system, and play a regulatory role for that system [39],[40].

Taken together, we propose the model of camptothecin toxicity in *S. cerevisiae* that RUB1-attachment of CUL3 would enhance the degradation of TOP1 cleavable complexes and, therefore, the blocking of RUB1-conjugation pathway could contribute significantly to increase the level of camptothecin toxicity in a cell growth as depicted in Figure 6B.

#### DNA Damaging-Agents: Cisplatin and MMS

MMS and cisplatin themselves are potent damaging agents. Genes of their sensitive strains are related to DNA repair system as expected (Table S2). In particular, all of GO terms related to DNA repair system resided in the top rank, but also had higher significant p-value and percentages in PC-constrained strains than those in PC-free strains (Table S2).

#### Microtubule Poisons: Nocodazole and Benomyl

Benomyl and nocodazole are known for microtubule poisons. As expected, tubulin-folding or binding-related terms were enriched. In particular, the GO term of Gim/prefolding complex resided in top rank only in PC-constrained strains. This complex is known for promoting formation of functional α- and γ-tubulin [43].

Those examples showed that the PC-based approach was able to highlight the subset of important genes in the relevant drug-target pathway more than PC-free approach. How can PC-constrained strains narrow down PC-free strains in this sensible way? A possible reason is as follows: some protein complex itself is composed of proteins representing specific biological function. In camptothecin and benomyl examples, PC181 is known for related ubiquitin activating protein complex, and PC413 known for Gim protein complex promoting functional microtubule formation (http://pombe.kaist.ac.kr/CMA/ModeOfAction.pl). By guilty-by-association, the set of genes of drug-sensitive strains specified by drug-sensitive complexes with specific biological function would be involved in the corresponding function although some of them are not severely sensitive to a given drug. It could give rise to enrichment of some of GO terms related to drug's cellular toxicity in PC-constrained strains.

Our PC-based approach would not properly work if too many numbers of insufficient and incorrect components of protein complexes are used as hidden factors to represent whole dynamic cellular events and also insufficient and incorrect biological interactions are integrated for the associations between PC and strains in our model. In that case, some true positives of drug-target genes would be lost in PC-constrained way, which could lead to too small a set of genes or a biased set of genes compared with true drug-targeted genes. It is well known that high throughput interaction data used in our model is incomplete in the sense that they contain a lot of false positives and false negatives. Nonetheless, our results showed that our model is robust against such deficiency in the data. The problem will be lessened as more and more high-throughput data become accumulated and validated.

In addition to the data quality of prior knowledge for our modeling, it should be noted that observed data for our modeling was not sufficient enough to represent drug's cellular toxicity on the genome scale. For the yeast *Saccharomyces cerevisiae*, each of the ∼6,000 potential genes characterized by the genome sequencing project has been deleted, identifying ∼1,000 essential genes and ∼5,000 viable deletion mutants. In current study, we used only chemical-genetic profiles of viable yeast haploid mutants as observed data for our modeling. It means that the effect of any essential genes to a bioactive compound was not able to be included as observations. To overcome this drawback, we will need to integrate observed data of diploid-heterozygous strains into our model. Heterozygous deletion strains allow the study of all 1,000 essential gene products. Furthermore, it was reported that drug-induced haploinsufficiency of diploid-heterozygote strains could discern the direct effect of bioactive compounds by assessing the consequence of reducing the amount of gene product [3],[4]. We believe that our PC-based modeling of chemical-genetic profiles can provide appropriate framework to deal with the combined chemical-genetic profiles of haploid and diploid-heterozygote strains as observation, and thus such extended model could especially contribute to predict more precisely relevant pathway including target-protein that interact directly with bioactive compounds.

### Conclusion

In a seminal article in the journal *Cell* entitled, “The cell as a collection of protein machines: preparing the next generation of molecular biologists,” Bruce Alberts described a cell as a factory: “Indeed, the entire cell can be viewed as a factory that contains an elaborated network of interlocking assembly lines, each of which is composed of a set of large protein machine” [9]. In recent genomewide study of yeast, two independent groups, European Molecular Biology Laboratory (EMBL), Cellzome (a spin-off company from EMBL), and the university of Toronto, have surveyed the first comprehensive protein complexes, called protein machines by Bruce Alberts, using tandem affinity purification (TAP) [8],[44]. Furthermore, Gavin et al. [8] gives molecular rationale of protein complexes for gene-to-phenotype relationship. In our study, those protein complexes are regarded as functional units for yeast cell growth, and then protein complex based Bayesian factor analysis is performed to relate growth fitness of genomewide deletion strains to hidden activities of protein complexes in a cell. In other words, at the organism's phenotype level, the chemical-genetic profiles representing relative growth fitness of systematic deletion strains are modeled in terms of protein complexes at the molecular levels.

To show that our model assumption (Figure 1) is reasonable and the inferred complex activities are reliable, hierarchical clustering analysis and literature survey were performed, which showed predictive power of common mode-of-action of bioactive compounds as well as grouping of protein complexes with similar biological behavior. In addition, we performed drug's target-pathway prediction based on our model assumption (Figure 1) to show how practical our model framework is. GO analysis and literature survey shows that complex-based way of drug's target-pathway prediction narrows down lots of drug-sensitive genes involved in various biological pathway to a handful of genes important in the relevant pathway perturbed by a drug. For example, we were able to highlight target-protein, TOR1, of rapamycin as well as RUB1, UBA3, UBC12, and ULA1 related to protein neddylation as relevant biological pathway for cellular toxicity of camptothecin.

From the purely computational standpoint, our model and Bayesian hidden component analysis (BHCA) developed by Sabatti and James [11] are essentially the same, as we pointed out in Method section. We did not try to improve the BHCA, even though there may be a number of ways to improve the algorithm itself, simply because that was not our main objective. Main focus of this study is to model the chemical-genetic profiles at the molecular level, more specifically using protein complexes, and we found that BHCA is an appropriate computational framework. The reasons are as follows: first, central assumption on a hidden factor is conceptually similar. The goal of BHCA in transcriptional regulation is to infer hidden activities of transcription factors under the assumption of combinatorial regulation of a gene by a set of transcription factors. Similarly, our goal is to infer hidden activities of protein complexes under the assumption of combinatorial effect of cell growth by a set of protein complexes. Under this assumption, we were able to set up BHCA-like Bayesian factor model, as shown in Figure 2. Key component of the model is **Z** matrix (binary association matrix), which not only allowed us to robustly compute the equation, but also provided a window through which relevant biological information such as protein-protein interactions and genetic interactions can be integrated with experimental data. Second, the estimation of protein complex activities by Bayesian factor model is more robust and stable because estimands are obtained by tens of thousands of samplings. Deterministic factor analysis methods [10],[12],[13] often give rises to numerically unstable solutions especially when improper prior knowledge is used in the analysis. In our modeling, protein complexes and genetic interactions as prior information are still insufficient and inaccurate, which makes it inappropriate to use deterministic methods such as network component analysis (NCA) for our study [10].

To improve the hierarchical clustering, Parsons et al. also utilized a matrix decomposition method known as probabilistic sparse matrix factorization (PSMF). Both PSMF and our factor model all decompose chemo-genomic profiles into some factors and their weights. These decompositions of the two methods contribute to noise-reduction of original data, and so reveal more essential features. For example, Parsons et al. discussed two cases (“factor 6” and “factor 5” in [5]) where PSMF method improved the results over hierarchical clustering. For our method, we have already illustrated a number of those examples in Results and Discussion Section. Moreover, if we compare the results from the strain-based clustering, PSMF, and the present method (PC-based clustering) all together, our PC-based clustering tend to produce the results that are more similar to those of PSMF than those of strain-based clustering (Figure S6), indicating that both decomposition-based methods represent some essential features through noise-reduction from original observation. What is different, however, is that our model uses known protein complexes as factors and fixed relationships between factors and observations, while PSMF infers both of them from observations. Therefore, our model is suitable when prior knowledge on factors and observations is available, and PSMF is suitable in case of no prior knowledge. Direct performance comparison between both methods is difficult because only four blocks in the whole “factorgram” are available from [5]. One distinctive advantage of our method over PSMF, however, is that because we use protein complexes as factors and integrate biological prior knowledge in our model, the inferred results are biologically interpretable, which allowed us to develop an effective way to predict drug's target pathway. In contrast, in PSMF biological meaning of factors is not clear. In addition, our model provides hidden activities of protein complexes from original data, which are difficult to measure in real wet experiment.

In current study, we used only chemical-genetic profiles of viable yeast haploid mutants as observed data for modeling. Consequently, it has a limitation for excluding the data for ∼1,000 essential genes in yeast. This drawback can be overcome by combining chemical-genetic profiles of homozygous and heterozygous deletion strains. We believe that our complex-based approach can provide appropriate framework for modeling such combined fitness data, which might especially contribute to predict more precisely relevant pathway including target-proteins that interact directly with bioactive compounds.

## Method

### Protein Complex (PC)-Based Bayesian Factor Analysis Model for Chemical-Genetic Profiles

Various types of factor models have been developed to model the transcriptional regulatory networks [10]–[13]. In factor models for transcriptional regulation, transcription factors are defined as hidden factors so that estimated effects of the factors can be biologically interpreted as the activities of transcription factors directly involved in the transcription. In a similar way, we developed a protein complex based Bayesian factor analysis (PCBA) for modeling chemical-genetic profiles. In essence, our model is similar to Bayesian hidden component analysis (BHCA) model by Sabatti et al. developed for the transcriptional regulation network analysis [11]. For convenience, we used the same notations and equations as described in the original BHCA paper.

The central assumption of our model was that the observed growth fitness measurements of each strain were determined by combined effects of the activities of a collection of the protein complexes (PCs) in each cell under different treatments. By log-transforming strains' fitness measurements, a linear model was formulated so that *e_it_* = *Σ^L^_j_*_ = 1_ a_ij_*p_jt_+*γ_it_*, where *e_it_* represented the relative fitness of a strain *i* in an experiment *t*; *a_ij_* the association strength of the protein complex *j* on the strain *i*; *p_jt_* the relative activity for the protein complex *j* in the experiment *t*; *L* the number of protein complexes as hidden factors; and finally *γ_it_* the measurement errors and the biological variability. We assumed that *γ_it_* was an independent and identically distributed Gaussian random variable following *N* (0, *σ* ^2^). If the number of strains is *N* and the number of experiments is *M*, the model can be rewritten in a matrix notation as follows:
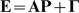
(1)


Here, **E** denoted an *N*×*M* matrix 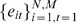. **A** was a matrix 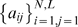 and represented unknown association strengths between strains and protein complexes. **P** denoted the matrix 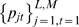.

In our model, there are the following features: (1) the “factors”, row vectors in the **P** matrix have a clear interpretation, as they correspond to specific protein complexes. (2) The specific value of the *p_jt_* is the primary interest for the prediction of drug's mechanism. (3) The matrix **A** is known to contain a large number of zeroes, corresponding to the sparseness of the network. This sparseness in our model is very important feature to solve the notorious non-identifiable problem in the factor model, by which multiple sets of different parameter values lead to the nearly identical likelihood. Therefore, the identifiability should be achieved by imposing some sort of constraints in the model. A typical way in biological analysis is to constrain the loading matrix of factors by the same size of the matrix with a specific pattern of zeros. In our factor model, the sparse network topology as such constraints was represented in **Z** matrix with the same size as the **A** matrix but with only 0 or 1 values (Figure S4).

The procedure for constructing **Z** matrix was shown in (A) and (C) of Figure 2. The entries, *z_ij_* were assumed to be independent. If there was the association between the knockout gene of a strain *i* and at least one of components in a protein complex *j*, we set *z_ij_* = 1. Otherwise we set *z_ij_* = 0. By letting *π_ij_* = *Pr*(*z_i_*_j_ = 1), we assigned 1 to the *π_ij_* because of limited memory size and computation time. By doing so, we lost the chance to remove false positive associations. The remaining entries of **Z** were set to zero. The other priors on **A**, **P** and *σ*|**Z** were also defined as follows:
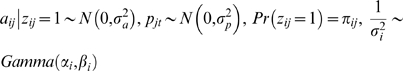
(2)The parameters *a_ij_*, *p_jt_* and 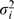 were assumed mutually independent. The priors on **A**, **P** and *σ* were mainly used for regularization. To explore the posterior distributions of the four parameter groups **Z**, **A**, **P**, and 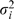, we used the collapsed Gibbs sampling based on the full conditional distributions of **Z**, **A**, **P**, and 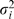, which were derived from those conjugate-like priors and joint probabilities, and described in the BHCA paper.

### Datasets for the PC-Based Bayesian Factor Model

We used the compendium of chemical-genetic profiles for 82 different bioactive compounds by screening them against the yeast haploid deletion collection, of ∼5,000 viable strains [5]. Each value in the profiles was represented by the combined average log2 ratio (control/experiment) of both barcodes (up tag and down tag) corresponding to each strain. We excluded multidrug-resistant genes [5], and genes of deletion strains not associated with any protein complexes. Finally, the profiles of 3,241 strains were used for our Bayesian factor model. We used the collection of 490 protein complexes (PCs) defined by the first genomewide characterization in an organism, budding yeast, using affinity purification and mass spectrometry [8]. The 488 PCs associated with at least one strains were used for our Bayesian factor model. For PC–strain associations, we used the BioGRID version 2.0.20 release containing physical and genetic interactions known in *S. cerevisiae* [45].

### Inference of Protein Complex Activities from the PC-Based Bayesian Factor Model

We obtained the posterior distributions of all the parameters of **A** and **P** matrices in our models using the collapsed Gibbs sampler derived in the BHCA paper [11]. The following hyperparameters were set for the Gibbs sampling: *α* = 0.7 and *β* = 0.3 for the gamma distribution as a prior of inverse 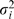, and *σ_a_* = 1, 10, 30, 60, 70, 90, 100 and *σ_p_* = 1, 10, 30, 50 for a priori standard normal distributions of *a_ij_* and *p_jt_*.

We first ran the Gibbs sampler for 11,000 iterations written in statistical language R [46] to select the optimal hyper-parameters, *σ_a_* and *σ_p_*. The initial 1000 iterations were discarded as burn-in, and the results of one per ten iterations were recorded, as suggested by Sabatti and James [11]. To assess the overall chain behavior, we monitored the sum of squared error (SSE). Given hyper parameters, *σ_a_* = 30 and *σ_p_* = 1, the convergence of SSE was best (Figure S5) so that we obtained multiple chains by Gibbs sampler under such hyper-parameter condition. For each chain of those multiple chains, we collected about 400 to 500 samples, one per ten iterations, after discarding initial 5,000 iterations as burn-in. All of samples obtained from each chain were merged so that they were used as each *posterior* (or sample) distribution of each parameter. Then, *posterior* means and *posterior* variances of each *posterior* distribution were used as estimands of each parameter. In this study, we focused on the analysis of *posterior* mean of each element in **P** matrix because our goal is to infer the hidden relative activities of protein complexes.

### Hierarchical Clustering Analysis

Hierarchical agglomerative clustering was performed by Gene Cluster 3.0 [47] for (A) and (D) of Figure 3 or by hclust function of standard R package stat [46] for (B) and (C) of Figure 3 using Pearson's correlation as a distance measure, and average linkage for compounds and complete linkage for protein complexes as an agglomeration method. The figures of clustering results were generated by Java TreeView [48] or by plclust function of standard R package stat.

### Selection of Significantly Sensitive Protein Complexes to a Bioactive Compound

To estimate the significance of the effect of a drug on a protein complex, we modified the error model used for haploinsufficiency-based direct drug-target identification in Yeast [4]. For this, first, the relative activities of all the protein complexes under different compounds were expressed as a matrix **P** with rows of 1…*i*…488 complexes and columns of 1…*j*…82 compounds, and the reference set of each complex was defined as a collection of activities of each protein complex under different 82 compounds, {*P_i_*_,*j* = 1‥82_}. For every activities (*P_ij_*) of a given protein complex under a given compound, the drug effect (*e_ij_*) on a protein complex was calculated by subtracting the mean (*X̅* *_i_*) of its reference set from *P_ij_*, and the uncertainty (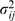) of the drug effect was obtained by pooling of the variance (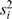) of its reference set and sample variance (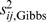) of *P_ij_* obtained from tens of thousands of Gibbs sampling (Equations 3 and 4).
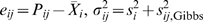
(3)

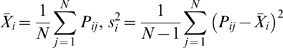
(4)where *N* represented the number of compounds, 82.

The drug effect (*e_ij_*) on a protein complex and its pooled variance (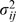) were applied to error function (Equation 5) so that significance of the effect of a given compound to a given complex was scored as a real value in the range of 0 to 1.
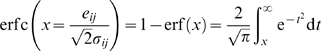
(5)When error function scores of complexes were less than 0.25, such complexes were regarded as being significantly affected by a given compound. When a protein complex has relatively positive value of activity, it was called a “sensitive complex”. In the opposite case, it was called a “resistant complex”.

In similar way, we defined “sensitive strain” and “resistant strain”, all of whose relative fitness values had greater than 0.5 or less than −0.5. Based on known biological associations between “sensitive/resistant complexes” and “sensitive/resistant strains”, Gene Ontology (GO) analysis for all of 82 compounds were performed, and those results were available at http://pombe.kaist.ac.kr/CMA/ModeOfAction.pl. Each set of genes of sensitive/resistant strains associated with sensitive/resistant complexes was used for GO analysis, which was also performed against all of genes of sensitive strains to compare complex-based with strain-based GO results in terms of highlighting relevant cellular pathway targeted by a compound.

## Supporting Information

Figure S1 The distribution of essential and non-essential genes in protein complexes. (A) The distribution of essential and non-essential genes involved in each protein complex is shown. The protein complex is composed of 5.5 essential and 7.8 non-essential genes in average. (B) All of 488 protein complexes include 1490 non-redundant genes, which consist of 580 essential and 910 non-essential genes. In particular, 52% of 1114 essential genes reported in Saccharomyces Genome Deletion project are included in 488 protein complexes.
(0.07 MB PDF)

Figure S2 Hierarchical clustering according to noise removal. (A) When chemical-genetic profiles of 4111 haploid strains just excluding non-viable and multi-drug sensitive strains were applied to hierarchical clustering, many of clusters are different from those obtained after 17% removal (Figure 5C in text). (B) When the relative activities of PCs lower than 0.015 and greater than −0.015 are set to 0, 133 of 488 PCs are removed (27% decreasing). Nonetheless, most of clusters are similar to those obtained before 27% removal (Figure 5B in text).
(0.06 MB PDF)

Figure S3 Two-dimensional hierarchical clustering. The set of the inferred protein complex activities was visualized by two-dimensional hierarchical clustering. In total, 82 compounds were clustered on the vertical axis, based upon the similar patterns of protein complexes, and 488 PCs were also clustered on the horizontal axis, according to the similar patterns of bioactive compounds
(8.71 MB PDF)

Figure S4 The row (strain)-wise and column (complex)-wise statistics of binary associations of **Z** matrix. The **Z** matrix comprises 488 columns (PCs) and 3241 rows (strains). The sparseness of **Z** matrix is shown in column- and row-wise counting. (A) The *x*-axis represents the number of strains associated with a protein complex. The *y*-axis represents the frequency of protein complexes with same number of associations with strains. The minimum, average, and maximum values of strains associated with a protein complex are shown in the histogram. (B) The *x*-axis represents the number of protein complexes associated with a strain. The *y*-axis represents the frequency of strains with same number of association with protein complexes. The minimum, average, and maximum values of protein complexes associated with a strain are shown in the histogram.
(0.05 MB PDF)

Figure S5 Plot of the sum of squared error (SSE). The chain of SSE sampled from Gibbs sampler seemed not to be sticky. It is used for monitoring overall convergences of parameters.
(0.02 MB PDF)

Figure S6 Comparison of grouping of drugs by probabilistic sparse matrix factorization (PSMF), protein complex (PC)-based hierarchical clustering and strain-base hierarchical clustering. The red arrow indicates drugs comprising a specific factor obtained by PSMF. (A) Group of drugs comprising factor 6. (B) Group of drugs comprising factor 5. The original factorgram images in the paper by Parsons et al. [5] were used in this figure.
(0.18 MB PDF)

Table S1 Functional annotations of protein complexes clustered together.
(0.02 MB PDF)

Table S2 GO analysis of drug-sensitive strains associated with drug-sensitive protein complexes.
(0.04 MB PDF)
