# Supplementary material for: Inference of Protein Complex Activities from Chemical-Genetic Profile and Its Applications: Predicting Drug-Target Pathways
Source: PLoS Comput Biol. 2008 Aug 29;4(8):e1000162. doi: 10.1371/journal.pcbi.1000162 (PMC2515108; doi:10.1371/journal.pcbi.1000162)
Supplement: Figure S1 — The distribution of essential and non-essential genes in protein complexes. (A) The distribution of essential and non-essential genes involved in each protein complex is shown. The protein complex is composed of 5.5 essential and 7.8 non-essential genes in average. (B) All of 488 protein complexes include 1490 non-redundant genes, which consist of 580 essential and 910 non-essential genes. In particular, 52% of 1114 essential genes reported in Saccharomyces Genome Deletion project are included in 488 protein complexes. (0.07 MB PDF) [file pcbi.1000162.s001.pdf]

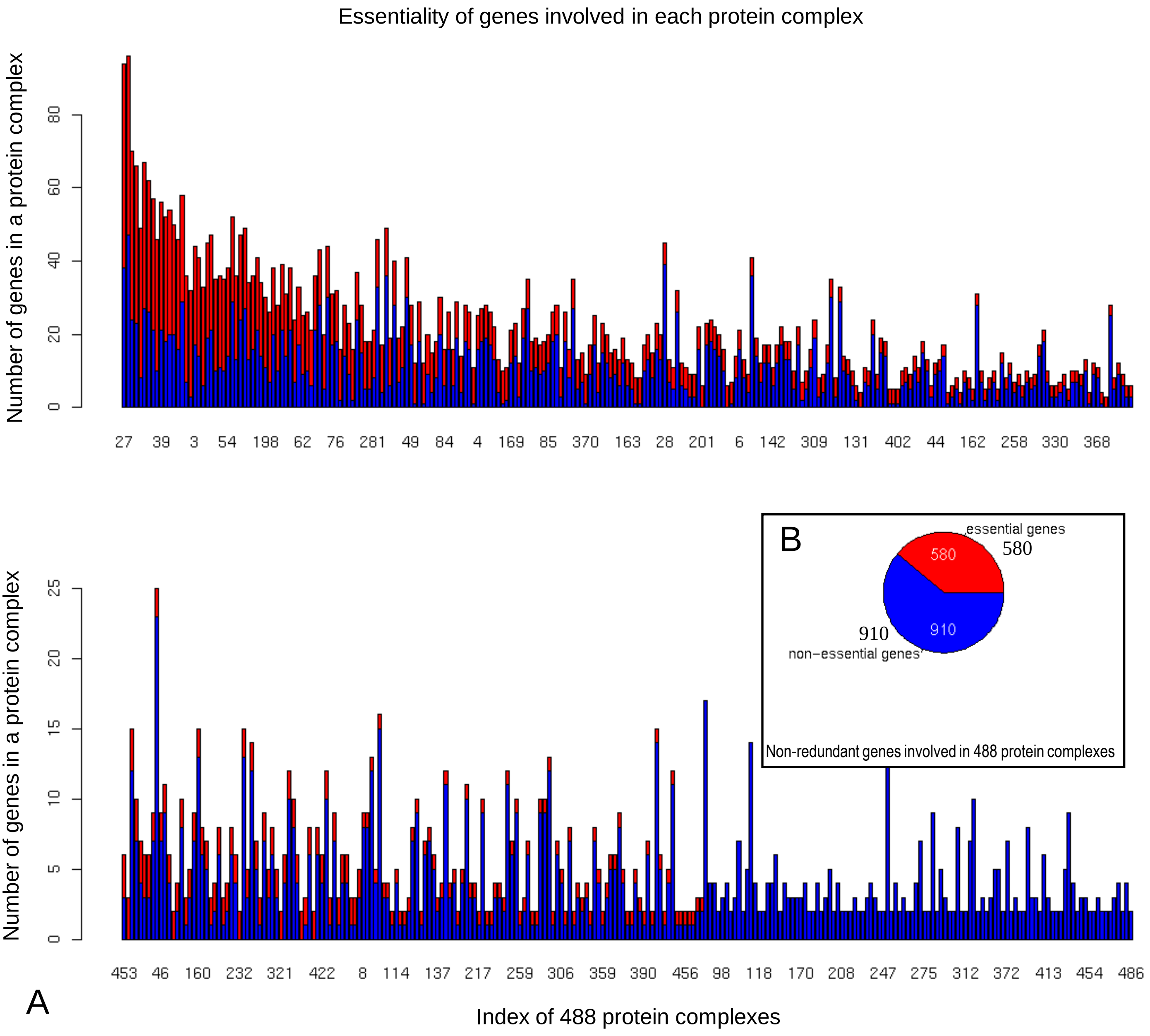

Figure S1 The distribution of essential and non-essential genes in protein complexes (A) The distribution of essential and non-essential genes involved in each protein complex is shown. The protein complex is composed of 5.5 essential and 7.8 non-essential genes in average. (B) All of 488 protein complexes include 1490 non-redundant genes, which consist of 580 essential and 910 non-essential genes. In particular, 52 % of 1114 essential genes reported in Saccharomyces Genome Deletion project are included in 488 protein complexes.
