# Supplementary material for: Inference of Protein Complex Activities from Chemical-Genetic Profile and Its Applications: Predicting Drug-Target Pathways
Source: PLoS Comput Biol. 2008 Aug 29;4(8):e1000162. doi: 10.1371/journal.pcbi.1000162 (PMC2515108; doi:10.1371/journal.pcbi.1000162)
Supplement: Figure S2 — Hierarchical clustering according to noise removal. (A) When chemical-genetic profiles of 4111 haploid strains just excluding non-viable and multi-drug sensitive strains were applied to hierarchical clustering, many of clusters are different from those obtained after 17% removal (Figure 5C in text). (B) When the relative activities of PCs lower than 0.015 and greater than −0.015 are set to 0, 133 of 488 PCs are removed (27% decreasing). Nonetheless, most of clusters are similar to those obtained before 27% removal (Figure 5B in text). (0.06 MB PDF) [file pcbi.1000162.s002.pdf]

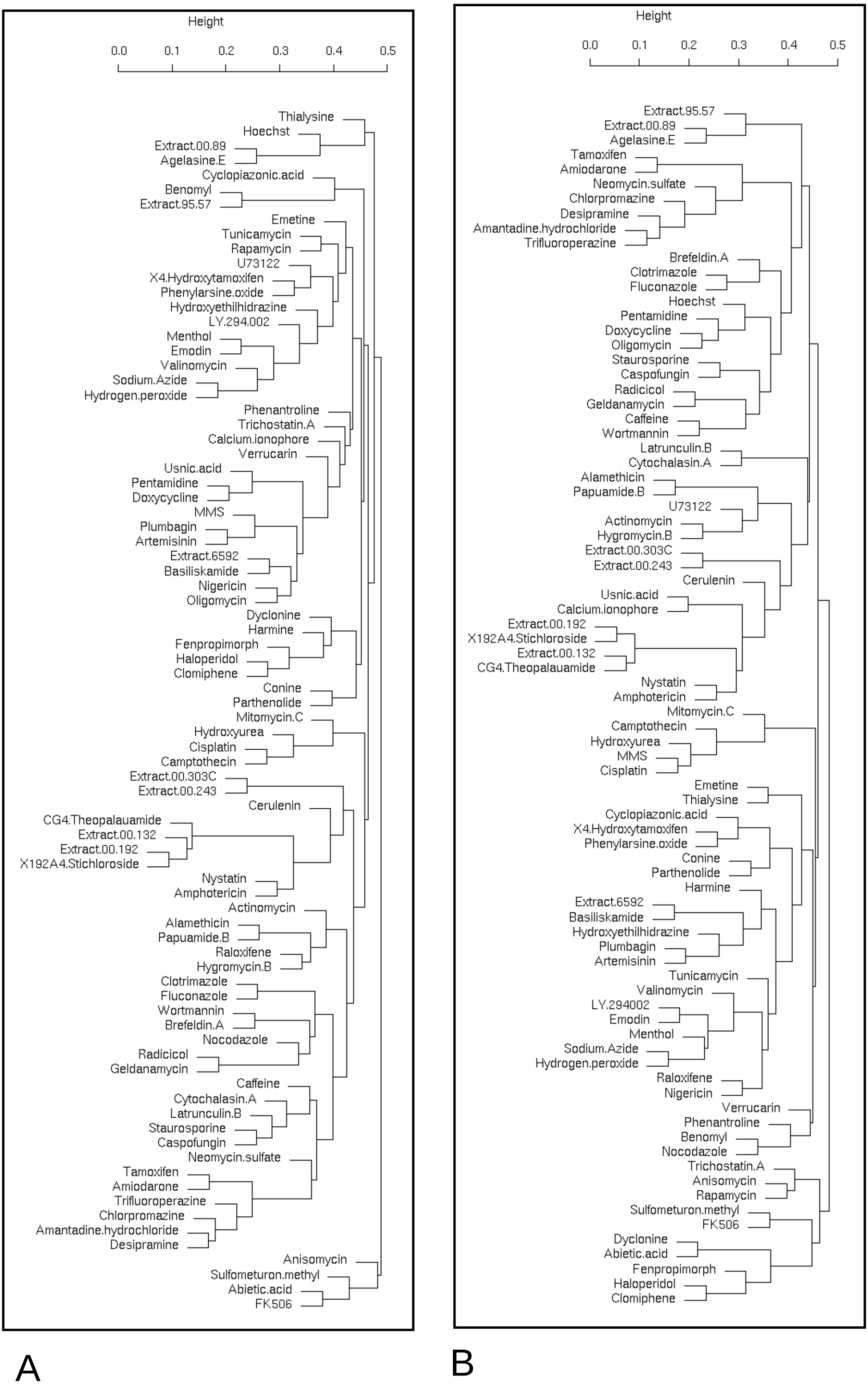

Figure S2 Hierarchical clustering according to noise removal (A) When chemical-genetic profiles of 4111 haploid strains just excluding non-viable and multi-drug sensitive strains were applied to hierarchical clustering, many of clusters are different from those obtained after 17 % removal (Fig. 5C in text). (B) When the relative activities of PCs lower than 0.015 and greater than -0.015 are set to 0, 133 of 488 PCs are removed (27 % decreasing). Nonetheless, most of clusters are similar to those obtained before 27 % removal (Figure 5B in text).
