# Supplementary material for: Inference of Protein Complex Activities from Chemical-Genetic Profile and Its Applications: Predicting Drug-Target Pathways
Source: PLoS Comput Biol. 2008 Aug 29;4(8):e1000162. doi: 10.1371/journal.pcbi.1000162 (PMC2515108; doi:10.1371/journal.pcbi.1000162)
Supplement: Figure S3 — Two-dimensional hierarchical clustering. The set of the inferred protein complex activities was visualized by two-dimensional hierarchical clustering. In total, 82 compounds were clustered on the vertical axis, based upon the similar patterns of protein complexes, and 488 PCs were also clustered on the horizontal axis, according to the similar patterns of bioactive compounds (8.71 MB PDF) [file pcbi.1000162.s003.pdf]

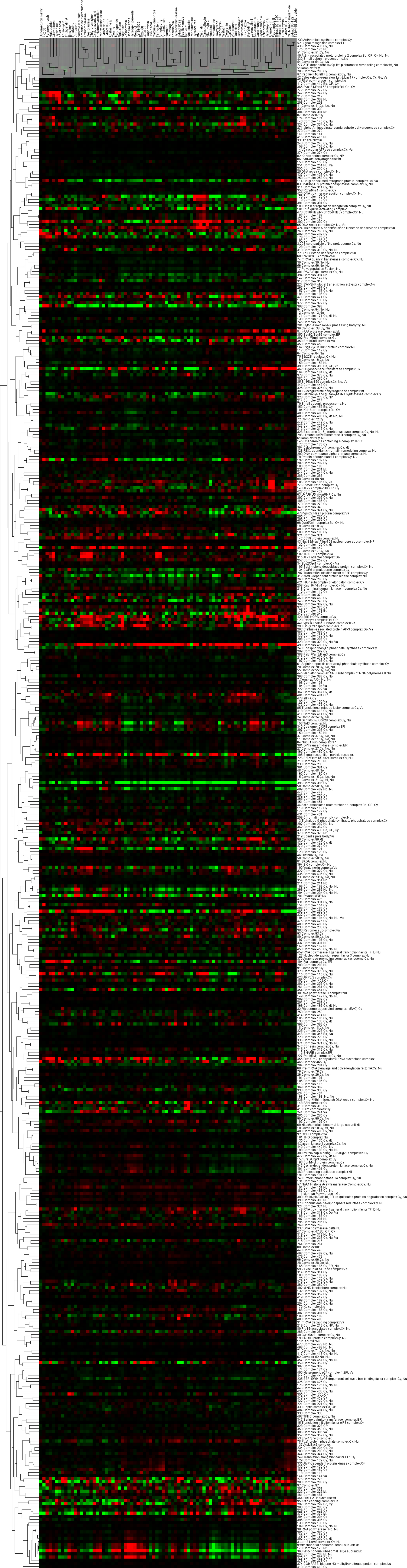

Figure S3 Two-dimensional Hierarchical clustering The set of the inferred protein complex activities was visualized by two-dimensional hierarchical clustering. In total, 82 compounds were clustered on the vertical axis, based upon the similar patterns of protein complexes, and 488 PCs were also clustered on the horizontal axis, according to the similar patterns of bioactive compounds.
