# Supplementary material for: Inference of Protein Complex Activities from Chemical-Genetic Profile and Its Applications: Predicting Drug-Target Pathways
Source: PLoS Comput Biol. 2008 Aug 29;4(8):e1000162. doi: 10.1371/journal.pcbi.1000162 (PMC2515108; doi:10.1371/journal.pcbi.1000162)
Supplement: Figure S4 — The row (strain)-wise and column (complex)-wise statistics of binary associations of Z matrix. The Z matrix comprises 488 columns (PCs) and 3241 rows (strains). The sparseness of Z matrix is shown in column- and row-wise counting. (A) The x-axis represents the number of strains associated with a protein complex. The y-axis represents the frequency of protein complexes with same number of associations with strains. The minimum, average, and maximum values of strains associated with a protein complex are shown in the histogram. (B) The x-axis represents the number of protein complexes associated with a strain. The y-axis represents the frequency of strains with same number of association with protein complexes. The minimum, average, and maximum values of protein complexes associated with a strain are shown in the histogram. (0.05 MB PDF) [file pcbi.1000162.s004.pdf]

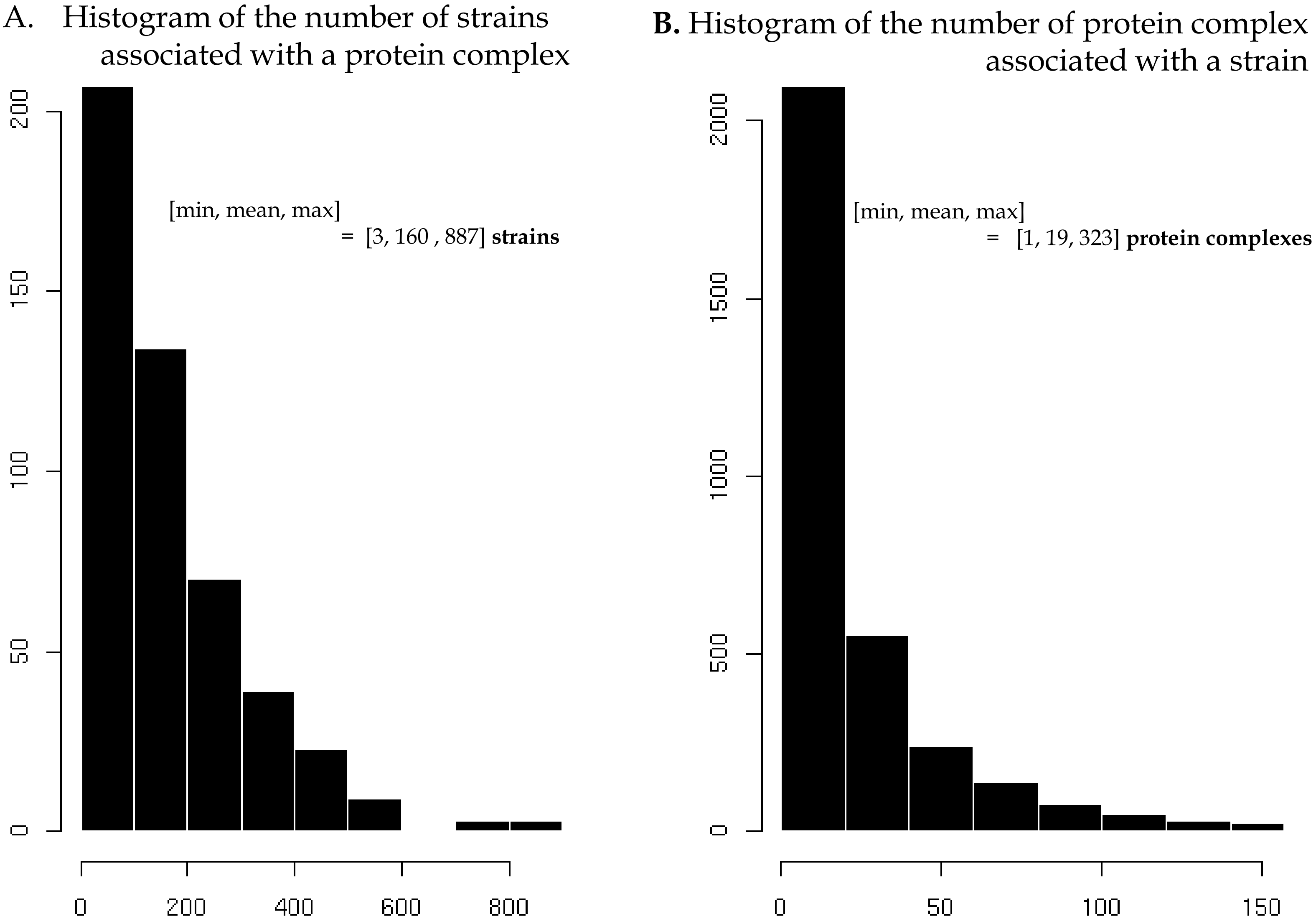

Figure S4. The row (strain)-wise and column (complex)-wise statistics of binary associations of Z matrix The Z matrix comprises 488 columns (PCs) and 3241 rows (strains). The sparseness of Z matrix is shown in column- and row-wise counting. (A) The x-axis represents the number of strains associated with a protein complex. The y-axis represents the frequency of protein complexes with same number of associations with strains. The minimum, average, and maximum values of strains associated with a protein complex are shown in the histogram. (B) The x-axis represents the number of protein complexes associated with a strain. The y-axis represents the frequency of strains with same number of association with protein complexes. The minimum, average, and maximum values of protein complexes associated with a strain are shown in the histogram.
