# Supplementary material for: Inference of Protein Complex Activities from Chemical-Genetic Profile and Its Applications: Predicting Drug-Target Pathways
Source: PLoS Comput Biol. 2008 Aug 29;4(8):e1000162. doi: 10.1371/journal.pcbi.1000162 (PMC2515108; doi:10.1371/journal.pcbi.1000162)
Supplement: Figure S5 — Plot of the sum of squared error (SSE). The chain of SSE sampled from Gibbs sampler seemed not to be sticky. It is used for monitoring overall convergences of parameters. (0.02 MB PDF) [file pcbi.1000162.s005.pdf]

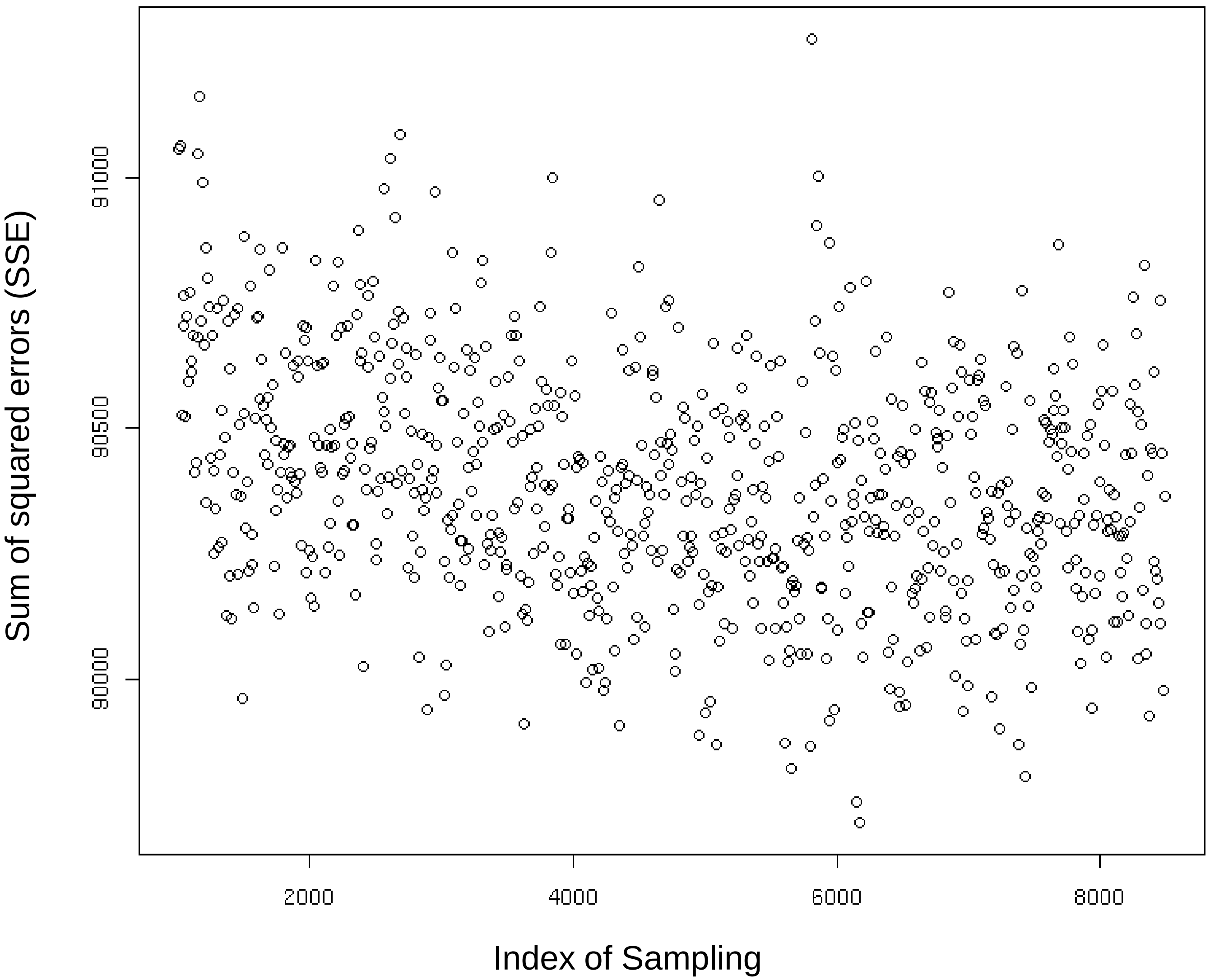

Figure S5. Plot of the sum of squared error (SSE) The chain of SSE sampled from Gibbs sampler seemed not to be sticky. It is used for monitoring overall convergences of parameters.
