# Supplementary material for: Inference of Protein Complex Activities from Chemical-Genetic Profile and Its Applications: Predicting Drug-Target Pathways
Source: PLoS Comput Biol. 2008 Aug 29;4(8):e1000162. doi: 10.1371/journal.pcbi.1000162 (PMC2515108; doi:10.1371/journal.pcbi.1000162)
Supplement: Figure S6 — Comparison of grouping of drugs by probabilistic sparse matrix factorization (PSMF), protein complex (PC)-based hierarchical clustering and strain-base hierarchical clustering. The red arrow indicates drugs comprising a specific factor obtained by PSMF. (A) Group of drugs comprising factor 6. (B) Group of drugs comprising factor 5. The original factorgram images in the paper by Parsons et al. [5] were used in this figure. (0.18 MB PDF) [file pcbi.1000162.s006.pdf]

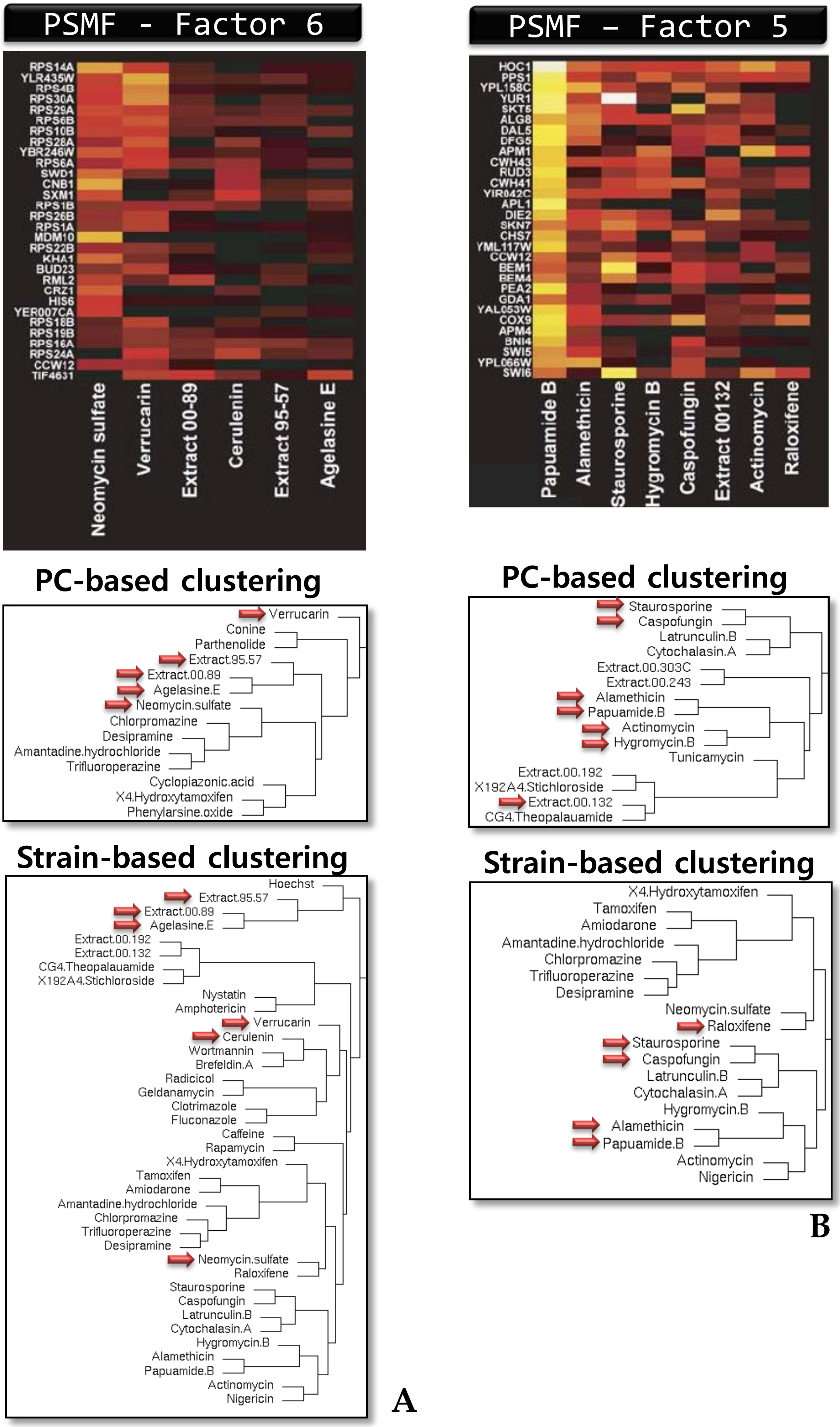

Figure S6 Comparison of grouping of drugs by probabilistic sparse matrix factorization (PSMF), protein complex (PC)-based hierarchical clustering and strain-base hierarchical clustering. The red arrow indicates drugs comprising a specific factor obtained by PSMF. (A) Group of drugs comprising factor 6. (B) Group of drugs comprising factor 5. The original factorgram images in the paper by Parsons et al [5] were used in this figure
