# Supplementary material for: Inference of Protein Complex Activities from Chemical-Genetic Profile and Its Applications: Predicting Drug-Target Pathways
Source: PLoS Comput Biol. 2008 Aug 29;4(8):e1000162. doi: 10.1371/journal.pcbi.1000162 (PMC2515108; doi:10.1371/journal.pcbi.1000162)
Supplement: Table S1 — Functional annotations of protein complexes clustered together. (0.02 MB PDF) [file pcbi.1000162.s007.pdf]

Table S1 Functional annotations of protein complexes clustered together

Cluster	Protein complexes (Protein complex ID)	Selected components	Functional annotations from SGD (Hirschman, Balakrishnan et al. 2006)
I	**Mitochondrial ribosome small subunit (PC 9)**	Overall	The smaller of the two subunits of a mitochondrial ribosome (Cellular Component, GO:00005763).
	**Mitochondrial ribosome large subunit (PC 363)**	Overall	The larger of the two subunits of a mitochondrial ribosome (Cellular Component, GO:00005762).
II	**HOPS complex (PC 429)**	overall	A multimeric protein complex that associates with the vacuolar membrane and is involved in homotypic vacuole fusion and vacuole protein sorting. For example, the Saccharomyces complex contains Vps41p, Vam6p, Pep5p, Vps16p, Pep3p, and Vps33p.(GO:0030897)
		Vps33	ATP-binding protein that is a subunit of the homotypic vacuole fusion and vacuole protein sorting (HOPS) complex; essential for membrane docking and fusion at both the Golgi-to-endosome and endosome-to-vacuole stages of protein transport; Golgi to endosome transport (GO:0006895), late endosome to vacuole transport(GO:0045324)
	**Clathrin-associated protein AP-3 complex (PC 262)**	Overall	An adaptor complex found associated with endosomal membranes; it is not clear whether AP-3 forms clathrin coats in vivo (GO:0030123)
		Apl5	Delta adaptin-like subunit of the clathrin associated protein complex (AP-3); functions in transport of alkaline phosphatase to the vacuole via the alternate pathway, suppressor of loss of casein kinase 1 function; Golgi to vacuole transport (GO:0006896)
		Apl6	Beta3-like subunit of the yeast AP-3 complex; functions in transport of alkaline phosphatase to the vacuole via the alternate pathway; exists in both cytosolic and peripherally associated membrane-bound pools; Golgi to vacuole transport (GO:0006896)
		Apm6	Mu3-like subunit of the clathrin associated protein complex (AP-3); functions in transport of alkaline phosphatase to the vacuole via the alternate pathway; Golgi to vacuole transport (GO:0006896)
	**Vps34p phosphatidylinositol 3-kinase complex (PC 441)**	Vps34	Phosphatidylinositol 3-kinase responsible for the synthesis of phosphatidylinositol 3-phosphate; forms membrane-associated signal transduction complex with Vps15p to regulate protein sorting; activated by the GTP-bound form of Gpa1p
		Vps15	Myristoylated serine/threonine protein kinase involved in vacuolar protein sorting; functions as a membrane-associated complex with Vps34p; active form recruits Vps34p to the Golgi membrane; interacts with the GDP-bound form of Gpa1p
		Vps30	Protein that forms a membrane-associated complex with Apg14p that is essential for autophagy; involved in a retrieval step of the carboxypeptidase Y receptor, Vps10p, to the late Golgi from the endosome; involved in vacuolar protein sorting
		Vps38	Part of a Vps34p phosphatidylinositol 3-kinase complex that functions in carboxypeptidase Y (CPY) sorting; binds Vps30p and Vps34p to promote production of phosphatidylinositol 3-phosphate (PtdIns3P) which stimulates kinase activity
	**Golgi transport complex (PC 293)**	Overall	A complex of proteins, that, in vitro, stimulates intra-Golgi transport; a 13S complex, about 800 kDa in size and consists of at least five polypeptides. In yeast, this complex is called the Sec34/35 complex and is composed of eight subunits (Sec34p, Sec35p, Dor1p, Cod1p, Cod2p, Cod3p, Cod4p, and Cod5p). (Cellular Component, GO:0017119)
		Cog1~Cog8	Essential components of the conserved oligomeric Golgi complex (Cog1p through Cog8p), a cytosolic tethering complex that functions in protein trafficking to mediate fusion of transport vesicles to Golgi compartments
	**Exocyst complex (PC 120)**	Overall	Protein complex peripherally associated with the plasma membrane that determines where secretory vesicles dock and fuse. At least eight complex components are conserved between yeast and mammals. (Cellular Component, GO:0000145)
		Sec3, Sec5, Sec6, Sec8, Sec10 and Sec15	The exocyst complex (Sec3p, Sec5p, Sec6p, Sec8p, Sec10p, Sec15p, Exo70p, and Exo84p), which has the essential function of mediating polarized targeting of secretory vesicles to active sites of exocytosis; ER to Golgi vesicle-mediate transport

Table S1 Functional annotations of protein complexes clustered together

**III**	**TRAPPII complex (PC 182)**	Overall	TRAPP (transport protein particle) complex; A large complex present on the cis-Golgi that acts prior to SNARE complex assembly to mediate vesicle docking and fusion (Cellular Component, GO:0030008); TRAPP is a multisubunit complex, and comes in two forms, TRAPPI and TRAPPII, which have different cellular localizations, cis-Golgi and trans-Golgi, respectively and also share a core of seven subunits. Furthermore, TRAPP I regulates the yeast Rab protein Ypt1 controlling the entry of cargo vesicles into the Golgi apparatus, and subsequently TRAPP II regulates Ypt31 and Ypt32 controlling the exit of the cargo from the Golgi (Morozova, Liang et al. 2006).
	**AP-1 adaptor complex (PC 315)**	Overall	An adaptor complex that links clathrin to the membrane surface of a vesicle; vesicles with AP-1-containing coats are normally found primarily in the trans-Golgi network. (Cellular Component, GO:0030121)
