# Supplementary material for: Inference of Protein Complex Activities from Chemical-Genetic Profile and Its Applications: Predicting Drug-Target Pathways
Source: PLoS Comput Biol. 2008 Aug 29;4(8):e1000162. doi: 10.1371/journal.pcbi.1000162 (PMC2515108; doi:10.1371/journal.pcbi.1000162)
Supplement: Table S2 — GO analysis of drug-sensitive strains associated with drug-sensitive protein complexes. (0.04 MB PDF) [file pcbi.1000162.s008.pdf]

Table S2. GO analysis of drug-sensitive strains associated with drug-sensitive protein complexes

Compound / Target pathway	Sensitive Complex ID	Number of sensitive strains	**Enriched GO terms (p < 10^{-4})**<br>**Detailed results are available at http://pombe.kaist.ac.kr/CMA/ModeOfAction.pl**
Rapamycin / **TOR pathway**	No complex	236	**Process:** transport (GO:006810, p<1.4e-9, 71 out of 232 genes(30.6%)), vacuolar transport (GO:000734, p<1.37e-11, 24 out of 232 genes(10.3%))
	PC 321	3	There is no enriched GO term. However, among three genes, TOR1 is PIK-related protein kinase and Rapamycin target as well as subunit of TORC1, a complex that controls growth in response to nutrients by regulating translation, transcription, ribosome biogenesis, nutrient transport and autophagy.
Camptotechin / **Topoisomarse I**	No complex	303	**Process:** response to DNA damage stimulus (GO:0006974, p <8.27e-25, 54 out of 296 genes (18.2%)), DNA repair (GO:0006281, p <3.94e-17, 41 out of 296 genes (13.9%)) , protein modification by small protein conjugation(GO:0032446, p < 0.00028, 15 out of 296 genes (5.1%))
	PC 181	5	**Process:** Protein neddylation (GO:0045116, p < 9.38e-12, 4 out of 5 genes (80%)), protein modification by small protein conjugation (GO:0032446, 4 out of 5 genes (80%))<br>**Function:** NEDD8 activation enzyme activity (GO:0019781, P <1.15e-06, 2 out of 5 genes (40%))
	PC 170	55	**Process:** response to DNA damage stimulus (GO:0006974, p <5.67e-25, 35 out of 53 genes (66%)), DNA repair(GO:0006281, p <3.48e-18,21 out of 53 genes (39.6%))

Table S2. GO analysis of drug-sensitive strains associated with drug-sensitive protein complexes

MMS / **DNA alkylating agent**	No complex	386	Less enriched than the result of the set of strains sensitive in Cisplatin
	PC 379	4	**Process:** pyruvate metabolic process (GO:0006090, p< 3.78e-06, 3 out of 4 genes(75%))<br>**Component:** mitochondrial pyruvate dehydrogenase complex (GO:0005967, p < 1.54e-8, 3 out of 4 genes(75%))
	PC 170	53	Similar to the result of the set of strains associated with PC170 in Camptothecin
	PC 424	19	**Process:** response to DNA damage stimulus (GO:0006974, p< 3.64e-20, 16 out of 19 genes (84.2%)), DNA repair (GO:0006281, p <6.92e-20, 16 out of 19 genes (84.2%)), double-strand break repair via single-strand annealing (GO0045002, p< 0.16e-13, 6 out of 19 genes (31.6%))<br>**Component:** nucleotide-excision repair factor 1 complex (GO:0000110, p< 3.58e-07, 3 out of 19 genes (15.8 %))
Cisplatin / **DNA inter- and intracross linking agent**	No complex	131	**Process:** response to DNA damage stimulus (GO:0006974, p< 1.53e-32, 44 out of 128 genes (34.4%)), DNA repair (GO:0006281, p< 7.74e-25, 35 out of 128 genes (34.4%)), double-strand break repair (GO006302, p< 1.44e-13, 16 out of 128 genes (12.5%)), double-strand break repair via single-strand annealing (GO:0045002, p< 7.31e-7, 6 out of 128 genes (4.7%))<br>**Component:** nucleotide-excision repair complex (GO:0000109, p < 0.0001, 6 out of 128 genes (4.7 %))
	PC 65	46	**Process:** response to DNA damage stimulus (GO:0006974, p< 2.10e-35, 31 out of 44 genes (70.5%)), DNA repair (GO:0006281, p< 2.4e-25, 24 out of 44 genes (72.7%)), double-strand break repair (GO:006302, p< 4.06e-16, 13 out of 44 genes (29.5%))<br>**Component:** Nucleotide-excision repair complex (GO:0000109, p < 5.3e-06, 5 out of 44 genes (11.3 %))
	PC 424	11	**Process:** response to DNA damage stimulus (GO:0006974, p< 1.53e-15, 11 out of 11 genes (100%)), DNA repair (GO:0006281, p< 6.35e-14, 10 out of 11 genes (90%)), double-strand break repair via single-strand annealing (GO:0045002, p< 2.41e-11, 5 out of 11 genes (45.5%))<br>**Component:** nucleotide-excision repair factor 1 complex (GO:0000110, p< 5.3e-08, 3 out of 11 genes (27.3 %))

Table S2. GO analysis of drug-sensitive strains associated with drug-sensitive protein complexes

Benomyl / **Microtuble-depolymerizing agent**	No complex	367	**Process:** tubulin folding (GO:0007021, p < 8.86e-07, 8 out of 359 genes(2.2%), 10 out of total genes)
	PC 379	3	There is no enriched GO term.
	PC 366	12	**Process:** protein import into nucleus (GO:0006606, p< 5.96e-05, 4 out of 12 genes (33.3%))<br>**Function:** oxidoreductase activity, acting on the aldehyde or oxo group of donors (GO:0016903, 3 out of 12 genes (25%))
	PC 413	45	**Process:** tubulin folding (GO:0007021, p< 4.27e-12, 7 out of 44 genes(15.9%), 10 out of total genes)<br>**Component:** prefoldin complex(GO:0016272, p< 1.15e-06, 4 out of 44 genes, 6 out of total genes)<br>**Function:** tubulin binding(GO:0015631, p< 1.53e-12, 8 out of 44 genes)
Nocodazole / **Microtuble-depolymerizing agent**	No complex	450	Similar to the result of the set of strains sensitive in Benomyl
	PC 413	59	**Process:** tubulin folding (GO:0007021, p < 3.90e-11, 7 out of 58 genes(12.1%), 10 out of total genes), M phase of mitotic cell cycle (GO:0000087, p< 7.54e-07, 11 out of 58 genes(19%))<br>**Component:** prefoldin complex (GO:0016272, p< 1.28e-08, 5 out of 58 genes, 6 out of total genes), spindle pole body (GO:0005816, p< 3.58e-05)<br>**Function:** tubulin binding (GO:0015631, p< 2.47e-11, 8 out of 58 genes)
	PC 148	7	Process: Tubulin folding (GO:0007021, p < 2.22e-06, 3 out of 7 genes(42.9%), 10 out of total genes)<br>Component: Prefoldin complex(GO:0016272, p < 2.58e-07, 3 out of 7 genes, 6 out of total genes), PAN complex(GO:0031251, p < 1.86e-05, 2 out of 7 genes(28.6%))<br>Function: Tubulin binding(GO:0015631, p < 2.47e-11, 8 out of 58 genes)
